# Vessel effects in organic chemical reactions; a century-old, overlooked phenomenon

**DOI:** 10.1039/d2sc01125e

**Published:** 2022-05-04

**Authors:** Michael Martin Nielsen, Christian Marcus Pedersen

**Affiliations:** Department of Chemistry, University of Copenhagen Universitetsparken 5 2100 Copenhagen O Denmark michaelnielsen@fas.harvard.edu cmp@chem.ku.dk

## Abstract

One of the most intriguing aspects of synthetic chemistry is the interplay of numerous dependent and independent variables *en route* to achieve a successful, high-yielding chemical transformation. The experienced synthetic chemist will probe many of these variables during reaction development and optimization, which will routinely involve investigation of reaction temperature, solvent, stoichiometry, concentration, time, choice of catalyst, addition sequence or quenching conditions just to name some commonly addressed variables. Remarkably, little attention is typically given to the choice of reaction vessel material as the surface of common laboratory borosilicate glassware is, incorrectly, assumed to be chemically inert. When reviewing the scientific literature, careful consideration of the vessel material is typically only given during the use of well-known glass-etching reagents such as HF, which is typically only handled in HF-resistant, polyfluorinated polymer vessels. However, there are examples of chemical transformations that do not involve such reagents but are still clearly influenced by the choice of reaction vessel material. In the following review, we wish to condense the most significant examples of vessel effects during chemical transformations as well as observations of container-dependent stability of certain molecules. While the primary focus is on synthetic organic chemistry, relevant examples from inorganic chemistry, polymerization reactions, atmospheric chemistry and prebiotic chemistry are also covered.

## Abstract 2

One of the most intriguing aspects of synthetic chemistry is the interplay of numerous dependent and independent variables *en route* to achieve a successful, high-yielding chemical transformation.

## Graphical abstract

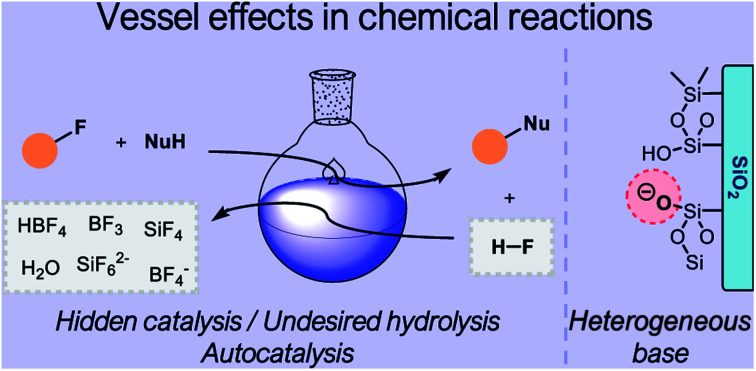

## Definition of vessel effects

The following review covers numerous examples of vessel effects observed during chemical reactions and during storage of chemicals. If a chemical reaction is affected (*e.g.*, product structure, yield, reaction time, reaction kinetics, mechanism or observed byproducts) by changing the vessel material whilst keeping all other reaction parameters unchanged we define the reaction as being subject to a vessel effect. Furthermore, the following review includes examples of container-dependent shelf life of certain chemicals, which is also included in our definition of a vessel effect.

This review includes examples from many fields of chemistry, which ranges from synthetic organic chemistry to inorganic chemistry and organometallic chemistry. It was not always possible to organize all the examples in well-defined and fully logical sections, but we have attempted to the best of our ability to make it coherent and at least provide fitting titles that encapsulate the essence of the individual sections. For more information on vessel material types, see Table 1 for an overview of the chemical composition.

**Table 1:** Overview of common vessel materials used for chemical synthesis

Common name	Material	Chemical composition or components	Functional residues
Pyrex®	Borosilicate glass	SiO_2_, B_2_O_3_, Na_2_O, K_2_O, Al_2_O_3_ (the composition varies between producers)	Silanols, silicates, boronates, water
Kimax®			
Duran®			
Jena®			
Soda-lime-glass		SiO_2_, K_2_O, Na_2_O, MgO, CaO, Fe_2_O_3_, Al_2_O_3_, TiO_2_, SO_3_	Silanols, silicates, metal oxygenates
Quartz		SiO_2_	Silanols, silicates
Teflon®	Polytetrafluoroethylene (PTFE)	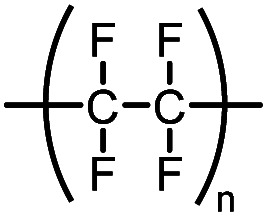	—
FEP	Polymer of fluorinated ethylene propylene	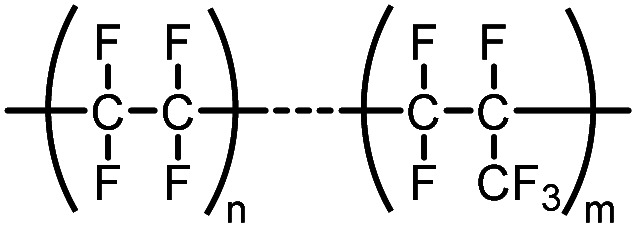	—
PFA	Polymer of perfluoroalkoxy alkanes	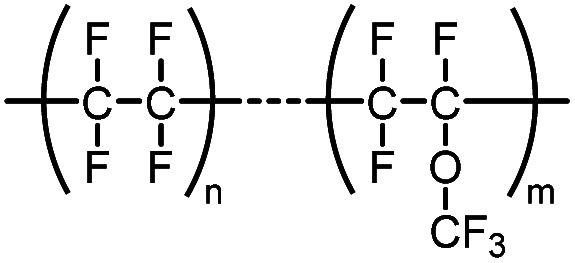	—
Kel-F®	Polychlorotrifluoroethylene	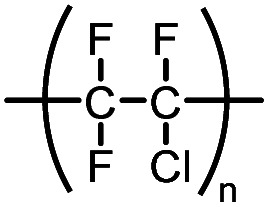	—
PP	Polypropylene	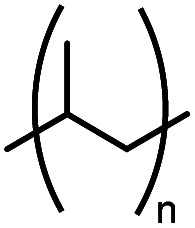	—
Stainless steel	Ferrous alloys	Iron, chrome, other metals	—
Monel	Nickel copper alloy	Nickel, copper, other metals	—

## Reaction vessel surfaces

Glassware is often regarded inert in chemical reactions and glass has therefore become the material of choice for laboratory reaction vessels. Over the years, several different types of glass have been used for making laboratory equipment for chemical reactions. Whereas soda lime glass and Jena® borosilicate glass found frequent use in the past, Pyrex®-type borosilicate glass is now by far the most used type of glass due to its chemical and thermal stability. Quartz reaction vessels have also found use in chemical laboratories but due to the significantly higher cost of manufacture of such vessels, this material is only rarely used for synthesis except for some photochemical reactions.

**Scheme 1:**
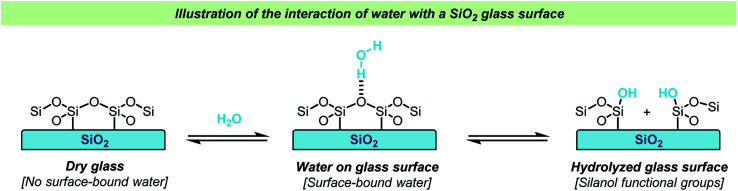
Simplified illustration of the adhesion of water to a glass surface and hydrolysis of a glass surface.

Glass vessels cannot generally be considered chemically inert. The most obvious reason for this non-innocence of glass vessels is the presence of water on the surface in an open-air lab environment (Scheme 1). This is something that most chemists are aware of, and careful drying of laboratory glassware is a routine operation in synthetic chemistry. The surface water content varies depending on the type of glass used and upon drying, the glass surface will still contain small amounts of water as well as silanols, boronates and aluminates depending on the glass type. Importantly, a glass surface is potentially chemically active post-drying and can take part in reactions, both as a promotor and as an inhibitor. Besides reactive functional groups on the surface, salts like Na_2_O are reported to diffuse from the glass into solution. Furthermore, this process is known to be dependent on the reaction conditions *e.g.*, the pH and solvent. A substantial amount of research in the field of material science has been dedicated to describing the chemical and mechanochemical properties of glass surfaces,^1–10^ but a detailed discussion of these aspects is out of the scope of this review.

A common method for deactivating the glass surface is to silylate the silanols in order to increase the hydrophobicity and electric resistivity of the surface.^11,12^ Silanizing or silylating glassware has become increasingly important when dealing with minute amounts of compounds, such as biomolecules, and therefore also relevant in biology and in the pharmaceutical industry.^13^ Standard procedures for chemical deactivation of the glass surface include using dichlorodimethylsilane, which gives short polymers on the glass surface, or trimethylsilyl chloride (TMSCl), to cap the silanols. The silylating reagents are normally dissolved in an organic solvent, which is subsequently removed by evaporation.^14^

The challenges associated with the chemical activity of standard laboratory glassware has made many chemists turn to fluorinated polymers like Teflon®, which is regarded as being chemically inert to most chemicals. However, recent studies have revealed that fluoropolymer-based membrane filters leach impurities.^15^ Additionally, Teflon® has been found to leach calcium ions, found to be a problem in chemical solution deposition for semiconductor film synthesis.^16^ Thus the choice of vessel material can have profound effects on a given chemical process and chemists should consider possible chemical interference from the chosen reaction vessel.

## Container-dependent stability of organofluorine compounds

**Scheme 2:**
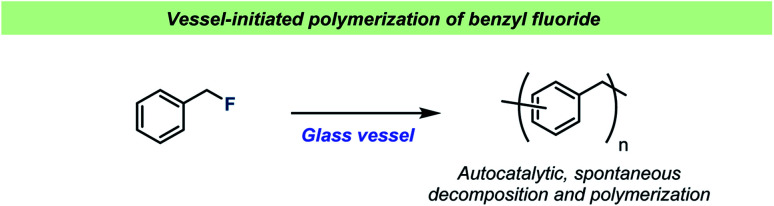
Spontaneous decomposition of benzyl fluorides in glass vessels reported by the Ingolds.^17^

For almost a century, the stability of certain organofluorine compounds has been known to depend on the container-material. This has especially been observed in studies involving organofluorine compounds capable of generating relatively stable carbocation intermediates upon departure of the fluoride leaving group. An early example was reported by the Ingolds in 1928 (Scheme 2) when encountering challenges during the synthesis and isolation of benzylic fluorides.^17^ They found that “*…benzyl fluoride decomposes spontaneously in glass vessels*”^17^ and that “*The reaction, which may develop with almost explosive violence, appears to commence at the glass surface*”^17^ leading to polymerization of benzyl fluoride. The Ingolds believed this process to be autocatalytic and found that the decomposition of benzyl fluoride was suppressed by using Jena® glass, a borosilicate glass type which contains sodium, magnesium and zinc.

Since the initial findings by the Ingolds, other researchers have made similar observations concerning the stability of organofluorine compounds of similar reactivity to benzyl fluoride. Bernstein *et al.*, reported that the synthesis of benzyl fluoride was very challenging and seemingly suffered from poor reproducibility.^18^ Accounts of challenges associated with the stability of organofluorine compounds are scattered in the scientific literature since the early reports by the Ingolds and Bernstein *et al.* Clark and co-workers made substantial efforts toward the synthesis of fluorinated metal hydrides in the sixties and reported several challenges associated with the stability of these.^19–22^ Clark reported that HF could eliminate from the metal complexes under certain conditions and proposed that “*The ready elimination of hydrogen fluoride, perhaps aided by the presence of silica but nevertheless occurring remarkably easily, can be attributed to the proximity of the hydridic proton to a fluorine atom in the olefinic complex*”^20^ and further noted that the elimination would be “*…accompanied by the formation of silicon tetrafluoride*.”^20^ This prompted a more in-depth study of the consequences of HF-elimination by the Clark team, leading to the discovery of BF_3_, BF_4_^−^, SiF_4_ and SiF_6_ being formed upon reacting with the glass vessel.^22^ Especially SiF_4_ was reported to increase the rate of elimination.^22^ Similarly, Kemmitt and co-workers have reported that carbonylrhodium complexes reacted differently with fluoroolefins depending on whether the reaction was taking place in a steel autoclave or in regular laboratory glass vessels.^23^ Cairns *et al.* reported spontaneous reactions with glass NMR tubes when trying to characterize fluorocomplexes of platinum, resulting in observation of NMR-resonances that are characteristic for silicon fluorides formed as a consequence of HF reacting with the glass surface.^24^ Related decomposition and reaction with glass vessels when handling fluorinated metal complexes has since been reported by Atherton^25^ and Gil-Rubio.^26^

An in-depth analysis of the difficulties associated with the isolation of neat allylic fluorides was made by Lee and Yandulov in 2009.^27^ They found that although several reports on the synthesis of such compounds existed in the literature, neat allyl fluoride would decompose within an hour in regular glass containers leading to the formation of polymers. They found that whereas Pyrex® glass containers (consisting primarily of SiO_2_ and B_2_O_3_ (ref. 27)) gave rise to rapid decomposition, soda lime glass containers (SiO_2_, Na_2_O and CaO/MgO as the major components) provided significantly longer shelf life for allyl fluorides. A polyfluoroalkoxy alkane (PFA) plastic container resulted in even longer shelf-life (>3 months in some cases), further emphasizing the vessel-dependent stability of the substance. Lee and Yandulov went on to characterize the by-products formed upon HF reacting with the glassware, identifying BF_3_, BF_3_·H_2_O, BF_4_^−^, SiF_4_, SiF_4_(H_2_O)_*n*_, SiF_6_^2−^, HF and HF_2_^−^ by NMR spectroscopy. Furthermore, it was argued that H_2_SiF_6_ and BF_3_(H_2_O) were more likely than HF to cause the autocatalytic decomposition of allylic fluorides as these are significantly stronger acids than HF. There is a considerable number of reports in the literature^28–37^ on the instability of allyl fluorides and certain alkyl fluorides when contained in glass vessels. Furthermore, it has been reported that attempted crystal growth of a gold bifluoride complex in a glass vessel led to the formation of an undesired complex bearing a pentafluorosilicate counterion.^38^ The desired bifluoride complex was successfully obtained by instead growing the crystals in a plastic vessel.

We mention here a vessel effect that does not fit into the following sections. Schreiber and co-workers reported that during a TBS ether-deprotection during the final steps of the total synthesis of FK-506,^39^ they found that using a polypropylene vessel the yield of the deprotection was increased to 73% instead of 30–35% yields when carried out in glass. The basis for this behavior was not investigated in detail, but it can be speculated that it is a consequence of the highly Lewis-acidic boron- and silicon fluoride byproducts (formed upon HF reacting with the glass vessel) causing either decomposition of the starting material or product.

## C–F bond activation

The activation of C–F bonds has received immense interest in the chemical community.^40^ The following section will highlight findings relevant to the descriptions of vessel effects during C–F bond activation reactions.

In 1952, a detailed kinetic study of the solvolysis of alkyl fluorides performed by Chapman and Levy^41^ concluded that the substitution of alkyl fluorides was autocatalytic when the reactions were initially neutral or contained less than 1 mol% HCl as a catalyst. Chapman and Levy believed that HF was the actual catalyst for the transformation and that the continuous production of HF was causing the autocatalytic kinetic profile as benzyl fluoride was expected to form hydrogen bonds to H_3_O^+^ or HF in solution.^41^ Chapman and Levy conducted a series of kinetic studies which led them to speculate that the consumption of HF by the glassware led to a lowered concentration of free HF (believed to be the active catalyst) and reported that HSiF_5_ and HBF_4_ were likely released into solution as HF reacted with the glass vessel. Furthermore, it was observed that certain alkyl fluorides would spontaneously and autocatalytically decompose upon stirring an ethanol solution in glass vessels indicating that even spontaneous decomposition of the alkyl fluoride (and consequent formation of HF in a protic solvent) was sufficient to initiate the following autocatalytic process.^41^ Coverdale *et al.*^42^ have reported that C–F bond activation was caused by an H-bonding event by HF in solution.

One of the useful features of alkyl fluorides is the possibility to perform C–F bond activation chemoselectively in the presence of other alkyl halide bonds using strong Lewis acids. This was described in the sixties by Olah and Kuhn^43^ who reported that BF_3_-promoted activation of primary alkyl fluorides occurred in the presence of analogous alkyl chlorides and bromides. The chemoselective activation of alkyl fluoride bonds in the presence of other alkyl halides has received considerable interest in the following years.^44–46^

**Scheme 3:**
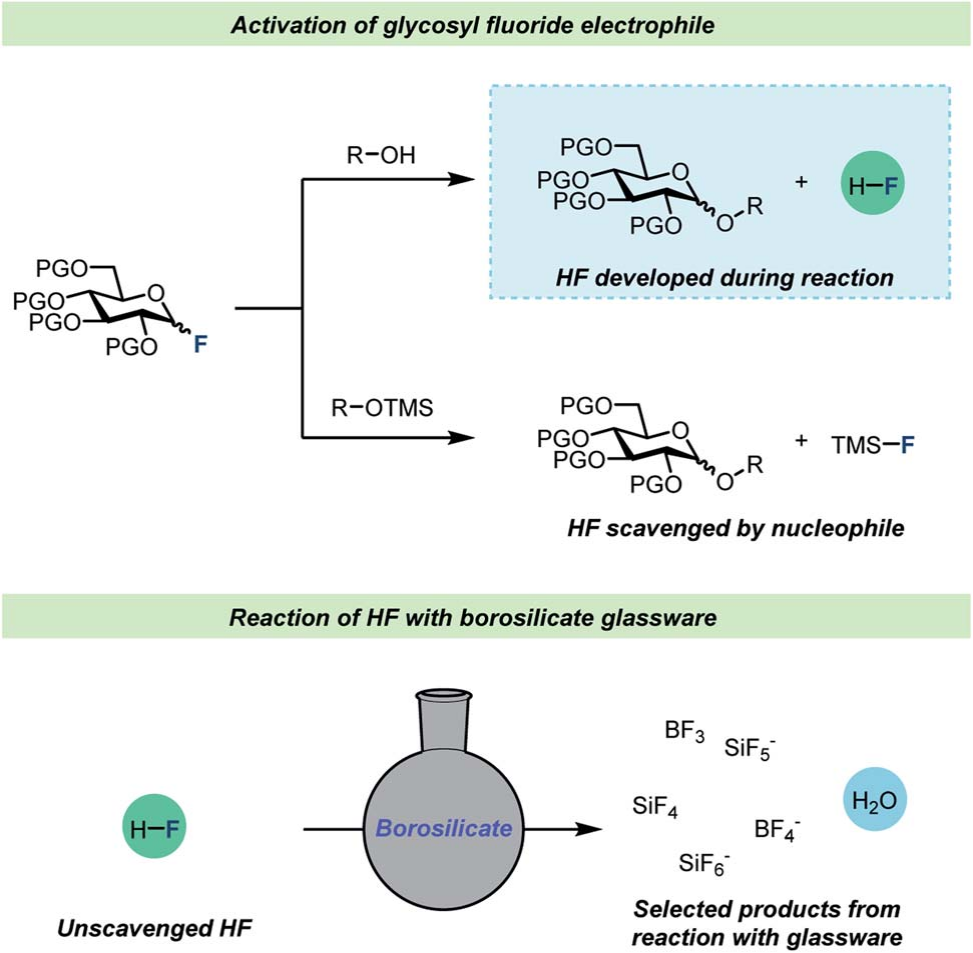
Top: reaction scheme for activation of glycosyl fluorides with either protic nucleophiles^52–57^ or silyl-protected^58–61^ nucleophiles. Bottom: selected products formed when HF reacts with borosilicate glassware. PG = protective group, L.A. = Lewis acid, TMS = trimethylsilyl.

One of the most well-studied examples of C–F bond activation chemistry is the activation of glycosyl fluorides during glycosylation reactions. Since the introduction of the fluoride leaving group in glycosylation chemistry by Mukaiyama and co-workers,^47^ glycosyl fluorides have become one of the most important and frequently employed electrophiles for catalytic glycosylations.^48–51^ Strong Brønsted and Lewis acids are employed for efficient glycosyl fluoride activation and a common feature for these glycosylations is that the use of desiccants,^52–57^ typically molecular sieves, seems to be pivotal for a successful glycosylation. There are two main strategies for glycosylations with glycosyl fluorides (Scheme 3): most commonly, protic nucleophiles are employed, but an alternative procedure involving silyl-protected nucleophiles (typically TMS-protected alcohols) has also found use. The most notable difference between the two strategies is the liberation of HF as the glycosylation with protic nucleophiles proceeds, whereas the glycosylations of silyl ethers^58–61^ avoid this.

**Scheme 4:**
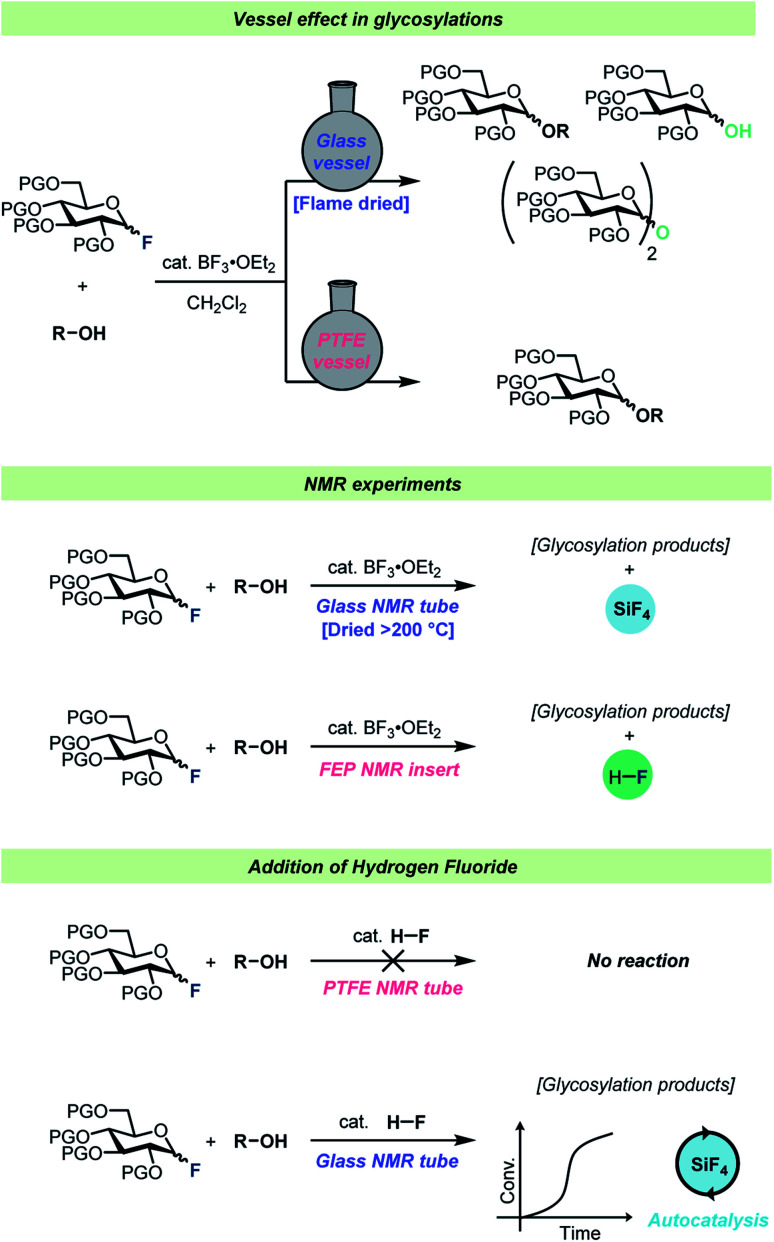
Summary of the observations by Wang, Pedersen, and co-workers.^62^

Interestingly, virtually no attention has been given to the fact that HF develops over time during the glycosylation of protic acceptors. To the best of our knowledge, the only consideration of this was by Kunz and Sager^61^ who added triethylamine to glycosylations of protic nucleophiles to neutralize the HF developed as the reaction progressed. Considering that HF is famous for its ability to etch glassware, it can seem somewhat surprising that such little attention has been paid to the formation of this species. To investigate whether the formed HF would in fact react with regular glass vessels, Pedersen, Wang and co-workers set up a series of identical glycosylations in either HF-resistant PTFE vessels or in regular borosilicate glass (Scheme 4).^62^ These experiments revealed that HF did indeed react rapidly with the glass vessels, resulting in the release of water as well as boron- and silicon fluorides into solution from the vessel. This vessel effect significantly impacted glycosylations of poor nucleophiles such as a 4-OH glycosyl nucleophile as this nucleophile was out-competed by adventitious water in the glass vessels, leading to poor yields of the desired product. On the other hand, the reactions of this weak nucleophile in HF-resistant vessels resulted in severe decomposition problems as the reaction medium became increasingly acidic as a function of fluoride consumption. Furthermore, reaction monitoring by *in situ* NMR, using standard borosilicate glass NMR tubes, and gas-phase GC-MS revealed that significant amounts of SiF_4_ were formed during glycosylations of glycosyl fluorides.

HF itself was found to be incapable of activating a glycosyl fluoride electrophile in a PTFE reaction vessel, however after spiking the reaction in borosilicate glassware with HF, the glycosylation reaction proceeded with an autocatalytic kinetic profile.^62^ The various boron- and silicon fluorides formed as the reaction progressed (all capable of activating the electrophile) were believed to be the origin of the autocatalytic kinetic profile.

**Scheme 5:**
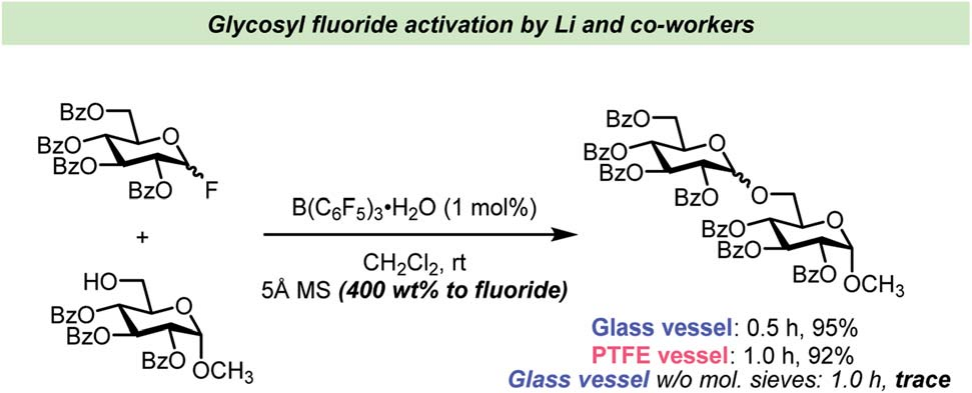
Control experiments for vessel effect by Li and co-workers.^63^ No experiment in PTFE vessel without mol. sieves was reported.

The report by Pedersen, Wang and co-workers^62^ has prompted other researchers to perform vessel-effect control experiments during glycosylations with glycosyl fluorides. Li and co-workers have recently introduced B(C_6_F_5_)_3_·(HF)_*n*_ (“BCF·(HF)_*n*_”) as an efficient catalyst for glycosyl fluoride activation^63^ (Scheme 5). In this report, the Li team concluded that the addition of 5 Å mol. sieves was crucial for the reaction, and that the reaction seemed to perform comparably well in glass and PTFE vessels as long as the desiccant was added. However, when carrying out the glycosylation in a glass vessel in the absence of mol. sieves, only a trace amount of the desired product was observed, which could be attributed to the hydrolysis of the electrophile by adventitious water released from the glass surface as reported by Pedersen, Wang and co-workers.^62^ A similar control experiment was unfortunately not undertaken in PTFE.

Turks and co-workers have recently identified SO_2_ as both a solvent and as an activator of glycosyl fluorides,^64^ which also gave rise to the discovery of a remarkable vessel effect. Whereas mannosylations proceeded in high yields at 100 °C in SO_2_, a comparable experiment in a PTFE vessel only resulted in 8% yield of the desired product and recovery of 53% of the electrophile. By increasing the reaction temperature to 150 °C, the yield in PTFE increased to 69%, but was accompanied by large amounts of a hydrolysis by-product. The hydrolysis is surprising as no water should be present in the reaction medium, nor should the PTFE vessel be capable of leaching water into solution as has previously been described for glass vessels. It remains unclear what the origin of the adventitious water was, but it seems that the most likely water-source is SO_2_, either as an impurity or *via* chemical decomposition of the solvent as the reaction proceeds.

**Scheme 6:**
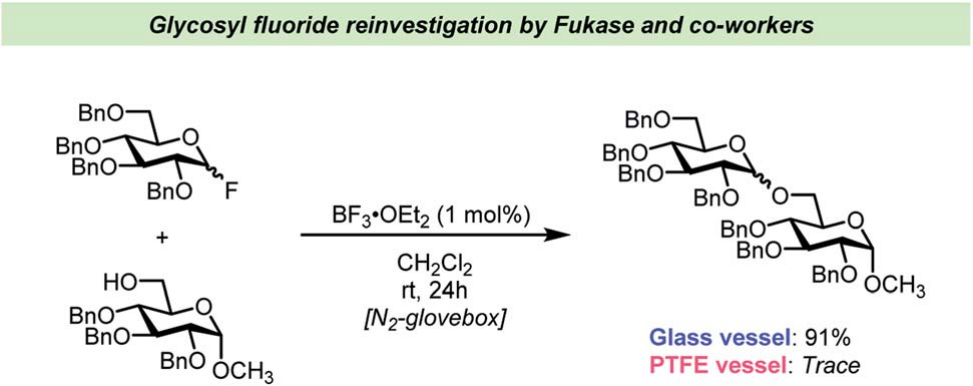
Selected example of the vessel effects observed by Fukase and co-workers.^65^

Very recently, a team of researchers led by Fukase reinvestigated the BF_3_·OEt_2_-catalyzed activation of glycosyl fluorides (Scheme 6).^65^ This study yielded some interesting findings on this otherwise very common glycosylation method. Not only did the team discover that as little as 1 mol% BF_3_·OEt_2_ was sufficient to activate both “armed” (highly reactive) and “disarmed” (relatively unreactive) glycosyl fluoride electrophiles, but that this was only the case in the absence of desiccants. The latter finding is remarkable as it is in contrast with most of the previous literature on activation of glycosyl fluorides (*vide supra*) that has generally relied on addition of large amounts of desiccants, typically 5 Å mol. sieves.

Furthermore, Fukase and co-workers confirmed^65^ the vessel effects described by the Pedersen team^62^ as it was found that the 1 mol% catalyst loading was only viable in glass vessels, whereas performing the glycosylations in PTFE vessels led to considerable decreases in yield. The Fukase team also reported that SiF_4_ formed as HF reacted with the glass surface and it was assumed that this was part of the catalytic cycle.^65^ It remains unclear how the release of water, which is a consequence of the reaction of HF with a glass surface, affects the reaction under the Fukase conditions and what the kinetics of this water release is. The recent studies on vessel effects during glycosyl fluoride activation shows that there are very apparent and seemingly important aspects of glycosylations using glycosyl fluorides that are yet to be resolved.

**Scheme 7:**
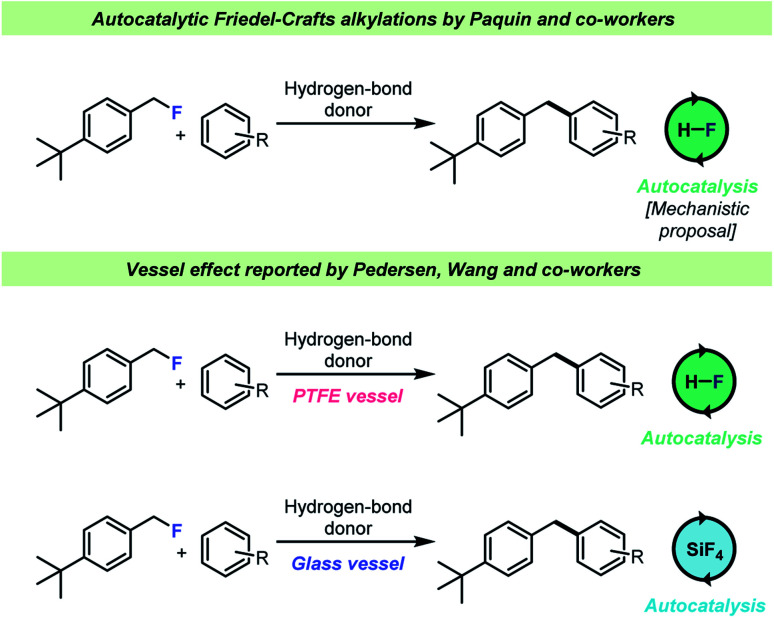
Vessel-dependent identity of the identity of the catalyst for Friedel–Crafts alkylations with benzyl fluorides.

The selective, catalytic activation of benzylic- and tertiary fluorides has attracted significant interest recently as it is possible to chemoselectively activate such C–F bonds in the presence of other halides. This reaction takes place under acidic conditions and has most notably been investigated by the Paquin^66–73^ and Moran^74,75^ teams (Scheme 7). An autocatalytic kinetic profile is commonly associated with these transformations^68,74^ which has been attributed to the fact that the HF (the leaving group in the cited publications) itself is capable of activating the C–F bond of the electrophile, *i.e.* by H-bond donation. This hypothesis was studied by Pedersen, Wang and co-workers^62^ who found that when a selected example^68^ of such a reaction was carried out in a borosilicate glass vessel, HF was immediately consumed by the vessel and instead the actual catalyst for this transformation was boron- and silicon-based Lewis acids formed by reaction with the glass surface (Scheme 7, bottom). However, when the reactions were conducted in an HF-resistant reaction vessel (an FEP NMR liner or PTFE vessels), the reaction still proceeded with an autocatalytic kinetic profile with HF as the actual catalyst, which confirmed the hypothesis by Paquin and co-workers.^68^ These results showed that the identity of the catalytic species in the autocatalytic C–F bond activation was dependent on the vessel material for the reaction.

The dependence on vessel material mandates that it is advisable to perform control experiments when developing methods for related reactions taking place under acidic conditions, with concomitant the formation of HF. An in-depth study on whether vessel effects generally affect the C–F bond activation reactions has not yet been undertaken, but it seems likely that some vessel effect could be in play.

Several reactions found in recent literature could potentially also be subject to some degree of vessel effect if the reaction is performed in glassware as HF seems highly likely to be formed as the reaction progresses. A comprehensive list of examples will not be given in the following as it is outside the scope of this review and somewhat speculative. We do however feel inclined to present a few representative examples of such reactions. Our objective is not in any way to criticize the work by the scientists, but rather to highlight some highly interesting and successful chemistry that theoretically could be influenced by the vessel material. One such example is an electrocatalytic S_N_Ar-reaction with aryl fluorides which was recently developed by Huang and Lambert.^76^ This reaction proceeds without the addition of base and should be expected to yield equimolar amounts of HF as the reaction progresses. The S_N_Ar procedure uses 5 equivalents of the aryl fluoride, which would allow for some of the electrophile to potentially be consumed by water released form the vessel surface without affecting the yield significantly, but no reaction optimization of the number of equivalents is presented in the paper and to the best of our knowledge, no experiment with one equivalent of the electrophile has been reported. Also, a recently developed catalytic difluoroalkylation reported by Wang and co-workers^77^ should also result in formation of one equivalent of HF during the final elimination step as only 105 mol% base is added, which allows for α-deprotonation of the reacting ketone, but not sufficient to sequester HF developed during the reaction.

It is still very unclear and widely undocumented what the implications of vessel effects during C–F bond activations are. However, given how easy it is to perform a vessel effect control experiment in practice (by simply switching otherwise optimal conditions to an HF-resistant vessel), we would recommend performing vessel control experiments during method development of C–F bond activation reactions under non-basic conditions.

## C–F bond formation

The field of C–F bond formation has been rapidly growing in recent years and important milestones in the development of selective fluorination reactions has been highlighted in several, in-depth reviews.^78,79^ However, as most C–F bond formations take place under basic conditions, the formation/persistence of HF should be suppressed and hence vessel effects of the same magnitude as described for C–F bond activation reactions above should not be expected. A few examples do however exist and will be presented in the following along with a discussion of possible avenues for investigation of vessel effects during C–F bond formation.

**Scheme 8:**
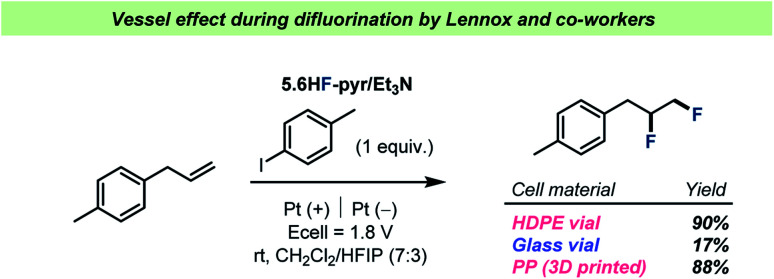
Performance of the three different electrochemical cell materials investigated for difluorination by Lennox and co-workers.^81^ HDPE = high density polyethylene.

Over the years, HF has been a frequently employed reagent in electrochemical reactions.^80^ However, as most commercially available electrochemistry equipment is produced from glass, researchers have had to rely on highly expensive, custom-made HF-resistant reaction vessels for electrochemical reactions with HF. To address this problem, the Lennox team recently reported the use of 3D-printed, HF-resistant reaction vessels for electrochemistry^81^ and showcased their effectiveness during a difluorination of olefins by pyridinium poly(HF) (Scheme 8). The reaction vessels were made from cheap, 3D-printable polypropylene (PP) and during comparisons with other reaction vessel materials, the team reported a significant decrease in yield when using an electrochemical cell made from glass (Scheme 8).

Lennox and co-workers argued that the drop in yield was due to consumption of HF by the glass surface, speculating that the formation of SiF_4_ upon reaction with the borosilicate glass surface could prevent the desired reaction. In any case, the results by Lennox and co-workers represent an example of a vessel effect during a reaction with poly(HF)–amine as a source of HF, a reagent that generally finds use in glass reaction vessels throughout the chemical literature.

As previously mentioned, most C–F bond activation reactions take place under basic conditions and thus should not be expected to be influenced by the choice of vessel material. However, the vessel effect reported by Lennox indicates that reactions involving poly(HF)–amine reagents could be subject to vessel effects in general. There are numerous examples of C–F bond forming reaction relying on such reagents taking place in glass vessels and it is much too speculative to discuss the absence or presence of a vessel effect across these examples. As a representative example of a very efficient and elegant C–F bond forming reaction, Doyle and co-workers recently reported a photocatalytic, nucleophilic fluorination using Et_3_N·3HF which takes place in glassware,^82^ but it has not been reported whether the choice of vessel material had any influence on the reaction. Nguyen and coworkers have previously shown that a quite similar formation of allylic fluorides was not influenced by either carrying out the reaction in glass or PP vessels,^83^ albeit using PP vessels in the standard procedure.

A rare example of a vessel effect during a deoxyfluorination reaction has been reported by Kucera and co-workers during a process development and scale-up of fluorinated proline derivatives.^84^ They reported that a deoxyfluorination with Deoxy-Fluor® which had been optimized in glass vessels gave just 10% conversion during scale-up in a polyfluorinated, HF-resistant 35 L reactor. It was believed that HF (formed by adventitious water reacting with Deoxy-Flour®) served as a co-catalyst in the deoxyfluorination, which led the team to add water to the reaction in the HF-resistant vessel. Unfortunately, this only increased the conversion to 30% after prolonged reaction time, which prompted a new hypothesis for the lowered reactivity in the HF-resistant vessel. In general, the deoxyfluorinations were faster and performed better when carried out in glass than in HF-resistant vessels indicating something else than just HF as the co-catalytic species.^84^ Kucera and co-workers realized that fluorosilicates formed upon deoxyfluorination of silica from the glass vessels were the actual co-catalyst for the deoxyfluorination, which led them to adding small amounts of silica to the reactions carried out in HF-resistant vessels. This led to a significant increase in the yield and the optimal scale-up procedure involved addition of 5 wt% SiO_2_ to the reactor.

It remains widely unknown how the vessel material can influence reactions that involve C–F bond forming reactions. Again, it seems to be pertinent to perform control experiments for a vessel effect when using poly(HF)–amine reagents or even when using common deoxyfluorination reagents such as DAST, Deoxy-Fluor®, XtalFlour-E® and XtalFluor-M®, *etc.* under non-basic conditions as free HF could be released into solution during such reactions.

## Vessel effects involving xenon difluoride and hypervalent iodine reagents

XeF_2_ is a valuable chemical reagent as it is a stable solid, which can be used a versatile fluorinating agent.^85,86^ Upon solvation, XeF_2_ turns into a very reactive reagent, which can react violently and even explosively with impure or wet solvents. Dukat *et al.* studied the reaction between XeF_2_ and various common organic solvents.^87^ The experiments were carried out in per-fluorinated ethylene propylene (FEP) tubes and monitored by NMR and it was found that CH_2_Cl_2_ decomposed faster upon reaction with XeF_2_ than solvents such as CHCl_3_ or MeCN. Ramsden and Smith later performed a comparative study and found that XeF_2_ could not even be detected when dissolved in CH_2_Cl_2_ or CHCl_3_ when instead using a regular glass NMR tube. XeF_2_ was simply found to decompose too quickly in the presence of a glass surface to allow detection by NMR. When dissolved in MeCN, the decomposition was slow and XeF_2_ could be studied by NMR using a regular glass NMR tube. The authors suggested that the weakly basic MeCN could neutralize acid on the glass surface and hence inhibit the catalytic decomposition.

**Scheme 9:**
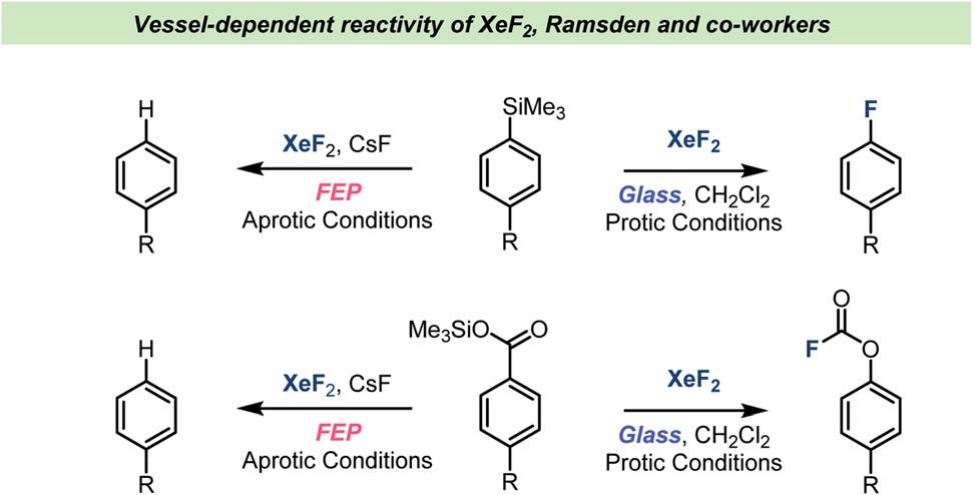
Selected examples of vessel-dependent reactivity of XeF_2_ reported by Ramsden and co-workers.

The Ramsden team have also investigated the XeF_2_-mediated formation and rearrangement of aryl fluoroformates (Scheme 9) and found that the reaction took place in halogenated solvents, but not MeCN.^88^ It was also demonstrated that aryltrimethyl silanes reacted with XeF_2_, resulting in formation of the corresponding aryl fluorides.^89,90^ Common for all these studies was that the reactions were investigated both in borosilicate glass vessels and in FEP vessels. Interestingly, the reactions were reported to not take place when using the more chemically inert FEP vessel material. Furthermore, glass vessels that were prewashed with NaOH solution were also found to inhibit the reactions. A glass surface with non-basic characteristics was therefore necessary for the fluorination reaction to occur. The importance of the vessel surface for the outcome of the reactions with XeF_2_ was demonstrated by performing several reactions under either aprotic conditions (FEP, NaOH washed glass or with MeCN, Scheme 9) or protic conditions (glass vessel and halogenated solvents). Under the aprotic condition, the active species was believed to be XeF_2_, which can react as a one-electron oxidizing reagent. On the other hand, when the protic conditions were used, XeF_2_ was reportedly ionized to XeF^+^, which was then the reactive electrophilic species. Hence, by choosing the right combination of vessel and solvent, distinct reaction paths could be favored, and specific products obtained with selectivity. Ramsden and Smith also performed reactions known to be dependent on presence of HF and showed that in glass vessels with chlorinated solvents, the reactions took place, which suggests that HF is formed when XeF_2_ is used with halogenated solvents in a glass vessel.

**Scheme 10:**
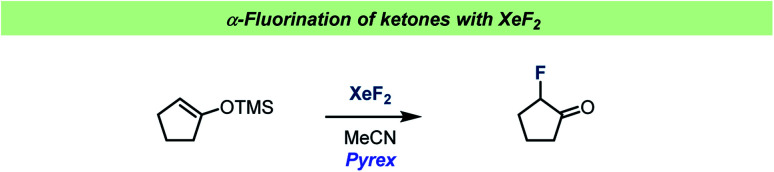
Conditions for α-fluorination of ketones by Ramsden and co-workers.^91^

The role of MeCN in reactions with XeF_2_ was further studied during reactions with silyl enol ethers (Scheme 10).^91^ Under aprotic conditions in glass a single electron transfer reaction took place, forming radicals, resulting in the formation of α-fluoroketones. When changing the solvent to halogenated solvents, under protic conditions, the reactions became more complex. This was attributed to the competing reactions involving XeF^+^. When the TMS enol ether of norcamphor was used, indirect evidence for a non-classic radical cation was obtained.

Ramsden and coworkers continued their studies of XeF_2_ in combination with different solvents and vessel materials. The reductive decomposition of CHCl_3_ was studied in more detail and was found to occur faster in Pyrex® glass vessels than in quartz suggesting that the Lewis acidic sites containing boron or aluminum take part in the reaction.^92^ The stability of XeF_2_ was greatest in FEP vessels, but the reagent was also very stable in glass vessels washed with NaOH prior to use. The latter observation was hypothesized to be a consequence of the base acting as a scavenger for Lewis acidic sites on the glass surface. The fluorodecarboxylation reaction was also revisited by Ramsden, this time directly from the carboxylic acid, upon reaction with XeF_2_.^93^ It was found that Pyrex® vessels worked as an effective heterogeneous catalyst for the reactions involving electrophilic XeF_2_ resulting in cyclizations, eliminations and rearrangements through reactions involving cationic intermediates. Reactions in PFTE vessels resulted in the decarboxylative fluorination. Patrick *et al.* had earlier performed similar reactions in polyethylene vessels, but apparently not studied the reactions in glass vessels.^94–96^

Ramsden and co-workers continued their systematic studies of vessel effects during the reactions of XeF_2_. NMR has been the primary analytical tool, but UV-Vis spectroscopy has also been used.^97^ The results have recently been described in an account reviewing their results in detail and giving an extended overview of the influence of vessel material and solvents on the outcome of reactions with XeF_2_.^98^

Lu and Pike synthesized [^18^F] xenon difluoride and studied it as a reagent for introducing ^18^F in organic compounds *via* fluorinations of silyl enol ethers (similar to Ramsden *et al. vide supra*). In line with the previous work, glass vessels were found to facilitate the reaction in contrast to polypropylene vessels that were reported to inhibit the reaction. MeCN was also found to be a less efficient solvent for this reaction when compared to CH_2_Cl_2_.^99^

The use of XeF_2_ in organic chemistry has been extensively studied, and often non-glass vessels have been used for these reactions, but prior to the work by Ramsden and co-workers, there were no systematic studies on the influence of the vessel material on this reagent. Some reactions are known to depend on initiation by HF, which is typically formed during many reactions of XeF_2_. As HF is known to etch glassware, HF-resistant perfluorinated plastics have typically been used as the standard vessels when handling XeF_2_ as guided by chemical intuition and the desire to preserve reaction vessels. Filler and coworkers used Kel-F® tubes for the reaction of XeF_2_ with substituted benzenes and disclosed that HF was important for the reaction to take place.^100,101^ Fedorov *et al.* used XeF_2_ in the presence of BF_3_·OEt_2_ in MeCN for the fluorination of aromatic compounds, but without specifying the reaction vessel used.^102^ Bardin and Adonin also studied the fluorination of aromatic compounds with XeF_2_ using BF_3_·OEt_2_ as the Lewis acid catalyst.^103^ Bardin and Adonin assumed that the reaction medium would be incompatible with regular glass vessels and instead HF-resistant FEP and PFA (block copolymer of tetrafluoroethylene and perfluoroalkoxytrifluoroethylene) vessels were used.

Gibson *et al.* have used glass vessels for studying several reactions with XeF_2_.^104^ The reaction of XeF_2_ with neat dimethylaminotrimethylsilane was peaceful until −35 °C. However, at −30 °C to −25 °C, a white solid formed followed by detonation, destroying the glass reaction vessel. A similar reaction with diethylaminotrimethyl silane resulted in a “*brisk reaction*”, which seemed to be independent of the vessel material used (baked out glassware, silylated glassware, Teflon® tubes, a monel reactor and polypropylene vessels were all tried). The reaction produces trimethylfluoride, Xe and diethylamine and no evidence for a radical mechanism could be found using ESR.

**Scheme 11:**
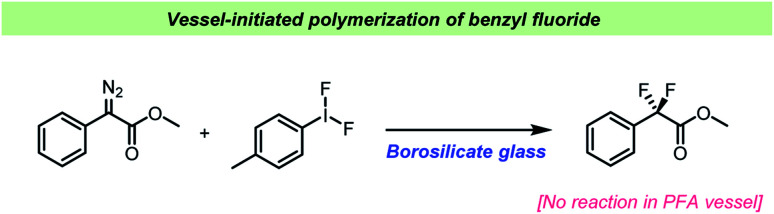
Vessel effects observed during difluorination by Sinclair *et al.*^107^

Despite the wide use of hypervalent iodine (poly)fluorides as fluorinating reagents, there are only few reports on the influence of the vessel used.^105,106^ Most often it is not specified and hence presumably, borosilicate glass is used. Sinclair *et al.* found that borosilicate glass could activate (difluoroiodo)toluene when used for *gem*-difluorination of phenyldiazoacetate derivatives (Scheme 11).^107^ Previous studies had used BF_3_·OEt_2_ as the Lewis acid catalyst for the *gem*-difluorination, but this caused problems when the substrates contained electron-rich functional groups, resulting in diminished yields.^108^ Sinclair *et al.* realized that the reaction took place without adding the catalyst suggesting that the reaction vessel could be involved.^107^ A computational study indicated that borosilicate is reasonably more Lewis acidic towards Tol–IF_2_ than BF_3_·OEt_2_. Based on these results, different reaction vessels and additives were studied, and it was found that the *gem*-fluorination only took place when borosilicate was present, either as the reaction vessel or as an additive in PFA vessels. Additionally, a reaction performed in silanized borosilicate glass vessel failed to yield the desired product.

## Other organic reactions influenced by the glass surface

In 1964 Dittmer and Mercantonio reported that the isomerizations of 1-chloro-2-butene and 3-chloro-1-butene were catalyzed by Pyrex® glass (borosilicate glass) and hence occurs during storage in laboratory glassware.^109^ The isomerization was however not observed when the compounds were stored in quartz vessels. A kinetic study revealed overall zero-order kinetics, which could suggest a very small concentration of free chloride ions as the driving force of the reaction. However, no free chloride ions or formation of butadiene could however be observed and there was no induction period, which should be associated with such a mechanism. The zero-order kinetics could also suggest a heterogeneous, catalytic reaction on the glass surface saturated with reactant, which would result in a pseudo-zero order reaction. The authors found this hypothesis most fitting to their observations, and boric oxide on the glass surface was proposed to be the actual catalyst of the reaction. A related isomerization reaction of isoprene catalyzed by HCl has been studied by Mascavage *et al.* who found that the reaction was in fact co-catalyzed by the Pyrex® glass surface.^110^ During this study, it was found that the reaction between HCl and isoprene involved surface-bound water-HCl species. In their study, an isomerization from 3-chloro-3-methyl-2-butene to the more stable 1-chloro-3-methyl-2-butene took place upon storage in glass vessels. Similar surface-catalyzed reactions involving alkynes and HCl have also been reported.^111^ It was found by Mascavage *et al.* that 2-butyne reacted to give (*Z*)-2-chloro-2-butene as the sole isomer. Kinetic studies have revealed that the reaction involves a surface-associated proton–alkyne interaction and that chlorine participation is not involved in the rate-determining step.^111^

**Scheme 12:**
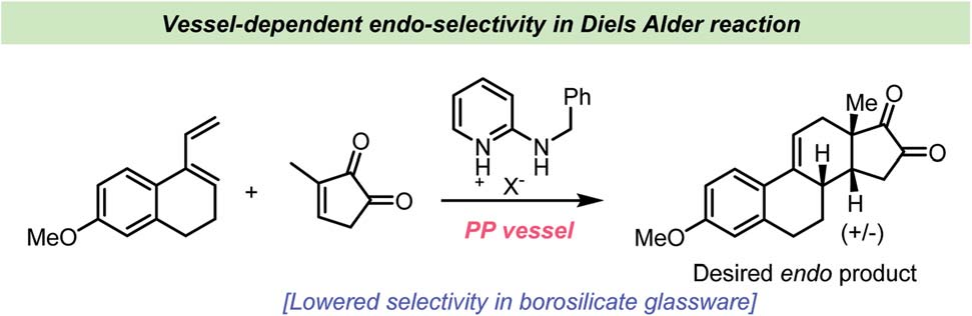
Example of vessel-dependent selectivity in *endo*-selective Diels–Alder reactions reported by Schuster *et al.*^112^

Schuster *et al.* studied the Diels–Alder reaction catalyzed by amidinium ions and demonstrated the use of this catalyst in the Quinkert-Dane estrone synthesis (Scheme 12).^112^ The addition of lipophilic amidinium ions favored the *endo*-selectivity required in the key intermediate towards estrone. During the study, it was revealed that special care had to be taken regarding the reaction vessel, as glass surfaces catalyzed the reaction as well and influenced the product ratio and yield. All reactions were consequently performed in polypropylene vials. The influence of silicates, such as chromatography absorbents, in Diels–Alder reactions had previously been described by Veselovsky *et al.*^113^

In an intramolecular Diels–Alder reaction, Reiser *et al.* found that standard glass vessels catalyzed the epimerization of the reactant, a trienolide. This could however be reduced by silylation of the glassware.^114^ The authors did not provide details for the epimerization and hence no mechanistic considerations were proposed.

**Scheme 13:**
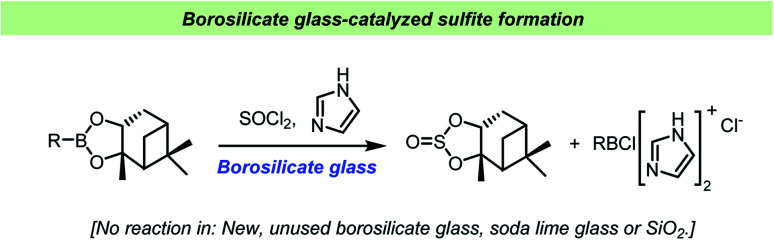
Vessel effect during sulfite formation reported by Matteson *et al.*^115^

Matteson *et al.* found that sterically hindered boronic esters reacted with thionyl chloride and imidazole on the surface of borosilicate glass to give the corresponding cyclic sulfite (Scheme 13).^115^ Interestingly, the reaction did not take place on soda lime glass surface, in the presence of silica gel or in new, unused borosilicate flasks, but initiated upon addition of borosilicate glass powder. Older flasks, regularly washed with potassium methoxide, were able to catalyze the reactions without addition of glass powder. This behavior further demonstrated how difficult it can be to predict the chemical implications of the vessel material as changes in cleaning procedures or even on the previous reaction history of a given reaction vessel can influence the outcome of a chemical reaction.

## Glass surface as a heterogeneous, Brønsted base-catalyst

Daley and Rodriguez found that the hydrolysis of silane coupling reagents used for glass-reinforced polymers were faster when soda lime glass was added to the reaction.^116^ The hydrolysis of the alkoxy silane reagent generates an intermediate which reacts with surface hydroxyl groups on the filler, *e.g.* glass fibers, which results in a reinforcement of the polymer. The hydrolysis reaction is known to be acid-catalyzed, but it was found that the presence of glass particles reduced the rate of hydrolysis. The presence of glass particles in the absence of acid, on the other hand, led to an increase in the reaction rate, which suggested that the glass surface was acting as a base catalyst. It could furthermore be demonstrated that the pH was slightly higher when glass was added to the acid-catalyzed reactions. The basic surface of the glass was therefore able to neutralize some of the acid. As it was found that vinyl silane was hydrolyzed faster with soda lime glass added than 3-(trimethoxysilyl)propyl methacrylate, it was suggested that steric effects play a role during the catalytic reaction at the glass surface. To study the influence of silanols present at the surface dimethylaminotrimethylsilane was used to inactivate the surface by silylation. The reaction rate was seemingly unaffected by the treatment. Crystalline silica gel was also used as an additive and had no effect on the rate, hence excluding the possibility that silanols were responsible of the catalysis. The authors proposed that alkali components from the soda glass leached into the solution and base-catalyze the hydrolysis reaction.

**Scheme 14:**
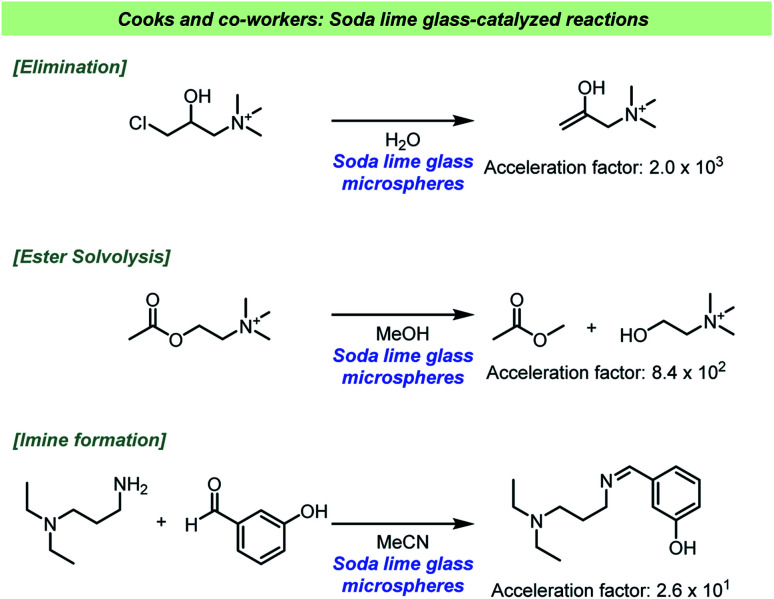
Examples of reaction rate acceleration in glassware reported by Cooks and co-workers.^117^

The ability for glass to act as a strong base heterogeneous catalyst has recently been further studied by Cooks and co-workers (Scheme 14).^117^ In a systematic study using ESI-MS and high-throughput experimentation, it became evident that various base-catalyzed reactions were catalyzed at the solid/solution interface. The reaction types found to be catalyzed included eliminations, condensations, oxidations and solvolysis reactions, which clearly indicates the broad scope of reactions potentially influenced by the choice of reaction vessel. The basicity of the surface depends on how the solvent interacts with the silanolates, which have greater base strength in aprotic solvents like MeCN. When the solvolysis of acetyl choline was performed in MeCN there was only minor effect, in contrast to the reaction in MeOH. Based on this, the authors suggested that the silanolate groups themselves are not nucleophilic, but only act as strong bases. As methanol is a protic solvent, methoxide is formed upon deprotonation by the glass surface, and it is the formation of methoxide that accelerates the solvolysis reaction. The glass microspheres could also be recycled, and it was confirmed that the catalytic reaction depended on the presence of the spheres and not ions or molecules leaching from the glass as the supernatant proven unable to catalyze the reactions. An interesting aspect of their study was base mediated degradation of biomolecules, such as phospholipids as well as the mentioned phosphocholine. Based on these findings storing such molecules in glass vessels should be avoided.

**Scheme 15:**
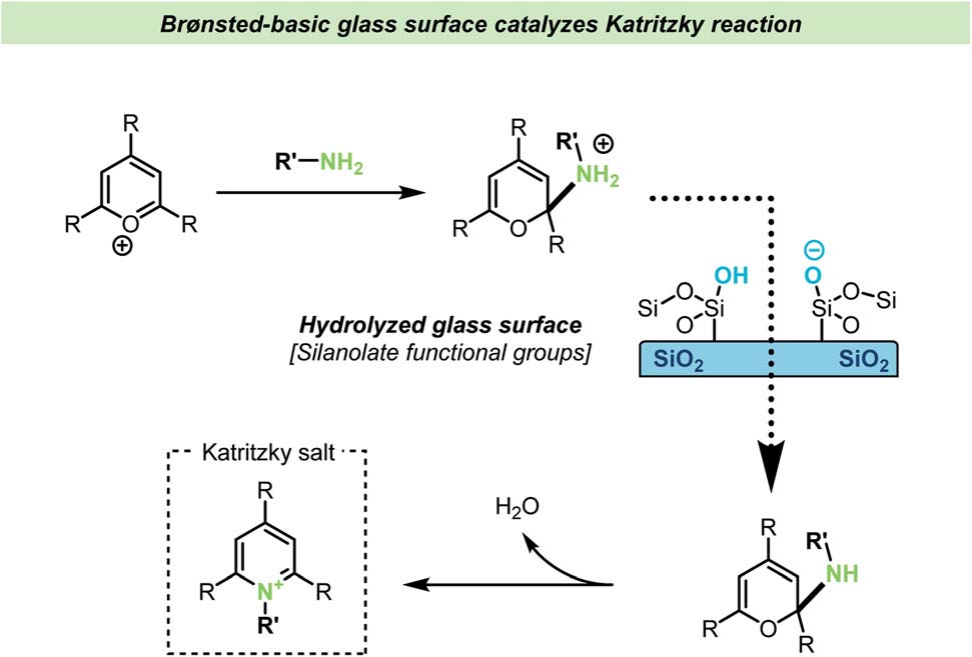
Brønsted-basic silanolate sites on a glass vessel surface were invoked to explain accelerated Katritzky reactions by Cooks and co-workers.^118^

In another very recent study by the Cooks group, the addition of lime soda glass particles was found to catalyze the Katritzky reaction.^118^ In this reaction a pyrylium salt reacts with an amine resulting in the formation of a pyridinium salt (Scheme 15). The reaction between the pyrylium salt and the amine takes place without the necessity of other reagents or catalysts, such as acid or bases. The reaction was studied in different reaction vessels, without prior treatment, and found to proceed at the lowest reaction rates in plastic vessels. It was found that new, uncleaned glass vessels gave faster reactions compared with cleaned ones (during the cleaning procedure, the inner walls of glass vials were triple rinsed with acetonitrile and allowed to dry). A series of control experiments showed that the presence of glass particles was important for achieving high reaction rates, but that the rate dramatically decreased if the glass particles were silanized prior to the reaction, suggesting that silanols on the surface of the glass particles are involved in the reaction. Further mechanistic studies showed that addition of one equivalent of acetic acid did not increase the reaction rate and yield, whereas addition of triethylamine increased the reaction rate to the same rate as the reaction containing glass particles. These observations together with the effect of silylating the glass surface support the hypothesis that the silanolate ions on the glass surface are responsible for the reaction acceleration by acting as a strong base. The glass particles were again found to be recyclable.

The production of biodiesel from cooking oil, *via* a base-catalyzed ester hydrolysis, using borosilicate glass catalysts has been demonstrated by Vadery *et al.*, who found that the actual catalyst was Na_2_SiO_3_.^119^ Similar work by Foroutan showed that waste glass could also be used as the catalyst for converting waste chicken fat in to biodiesel.^120^ In both studies the glass had to be treated with NaOH to become an active catalyst. This indicates that the basicity of the glass could be important as well.

In a groundbreaking experiment dating back to the early 1950's Miller and Urey found that organic molecules were formed when a mixture of methane, ammonia, hydrogen and water were exposed to electric sparking.^121,122^ Criado-Reyes *et al.* have followed up on the experiment and found that borosilicate glass plays a decisive role for the outcome.^123^ By comparing the reaction in Teflon® vessels and in Teflon® vessels containing small pieces of borosilicate glass to the original experiment carried out in a borosilicate vessel, they were able to demonstrate that the presence of glass clearly affected the reaction. In the presence of borosilicate glass, a dipeptide, dicarboxylic acids, polycyclic aromatic compounds and several biological nucleobases were formed exclusively, or to a greater extent, compared to the reaction in a Teflon® vessel. The surface of the reactor played a decisive role for the outcome of the reaction and an organic film could be observed on the glass surface above the water. These results point towards the importance of inorganic solids for the formation of biologically important compounds.

Carbohydrate synthesis *via* the formose reaction has also been found to depend on the presence of minerals closely related to the those found in glass.^124^ The exact role of borosilicate glass in this particular reaction remains unclear, posing a potentially interesting further investigation.

## Polymerization reactions catalyzed by glass

Moustafa and Diab studied the polymerization of methyl methacrylate using sodium bisulfite as the initiator and found that the rate of polymerization increased with the amount of soda lime glass added.^125^ Interestingly, it was also found that the polymer yield increased with the glass particle size used. Sodium bisulfite only slowly initiated the polymerization of styrene and failed to polymerize acrylonitrile, but in the presence of soda lime glass, both polymerizations were promoted. The authors proposed that the reaction depended on the formation of an addition product between sodium bisulfite and the soda lime glass to generate the active catalyst. When the polymerization of methyl methacrylate was carried out using colored glass (soda lime amber glass, which contains a small amount of carbon), the rate of polymerization increased further.^126^ In the absence of glass, the average molecular weights of the polymers were higher, but the conversion of monomer lower. With soda lime glass present it was observed that the larger grain size resulted in lower conversion, but higher average molecular weights.^127^ The polymerization of methyl methacrylate was also demonstrated to occur using sulphur dioxide in the presence of calcium sulfite or natural sand, but not in the absence of these inorganic substances.^128^

**Scheme 16:**
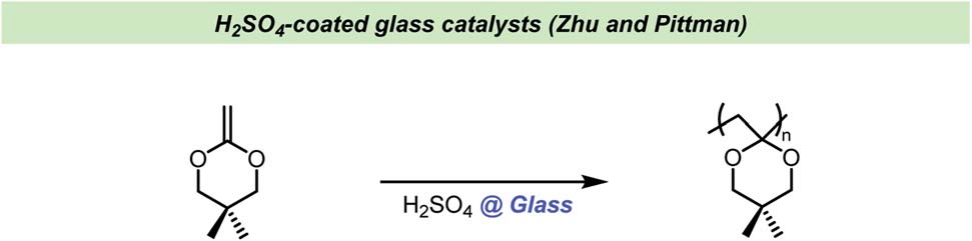
Zhu and Pittman using H_2_SO_4_-coated glass pearls.^129^

Zhu and Pittman found that glass beads coated with dilute H_2_SO_4_ and dried could catalyze the cationic polymerization of cyclic ketene acetals (Scheme 16).^129^ Using these coated glass pearls did not result in adventitious acid in the produced polymers, however it was difficult to control the acid strength and therefore the authors turned to acidified activated carbon. In this study, all glassware was washed with KOH/iPrOH and rinsed with water before use, but no exact explanation for this pretreatment of the reaction vessels was given. The treatment of glassware in a base bath could potentially result in cation exchange in the glass, allowing a build-up of potassium salts in glassware over time. An indication as to why this pre-treatment was important has been given by Crivello *et al.* who observed that ketene acetal monomers underwent thermally induced polymerization upon standing in glass container and that the glass surface catalyzed this reaction.^130^ The spontaneous polymerization could be avoided by washing the glass vessels with aqueous base prior to use or simply by adding potassium carbonate or potassium hydroxide to the monomers stored in glass vessels.

## Inorganic reactions at the glass surface

It has been known for almost a century that Pyrex® glass (borosilicate glass) surfaces can absorb small amounts of small acids like HCl. This was first studied in detail by Boggs and Mosher^131^ who were able to demonstrate that HCl indeed reacts with glass and electron microscopy could reveal that the otherwise smooth glass fibers became rough and uneven. A kinetic study revealed that the extent of reaction depended on the temperature and that the reaction rate of HCl reacting with the glass surface was diffusion controlled. The reaction could either be dependent on HCl diffusing into the glass, Na_2_O diffusing from the glass into solution, or dissolution of HCl in a layer of water on the glass surface. Boggs *et al.* continued their study with other acidic gases and found that HBr also reacts with Pyrex® glass, whereas H_2_S, SO_2_ and CH_3_Cl did not react at a measurable rate.^132^ It was found that raising the temperature to 450 °C gave irreproducible results, which could be explained by a mechanism where the gas diffuses into a water film on the glass surface. At high temperatures, this water film cannot be maintained, and the rate of diffusion decreases. Miyahara and Tsumura have reported that the equilibration of hydrogen at elevated temperatures (protium and deuterium exchange) was highly dependent on whether quartz, soda lime or borosilicate glassware was used.^133^ Based on this study it was suggested that the glass surface itself was involved in the reaction mechanism. Contaminants in the glass as well in the gas were also found to influence the reaction and catalytic properties of the glass. It is however still unclear exactly how the glass surface is involved in the reaction mechanism.

Winter *et al.* studied the interchange of O^18^ between water and inorganic oxy-anions.^134^ Their study was initiated by the suspicion that soluble silicate from the glass vessel could interfere with the reaction and they therefore used a silver container for the experiments. The exchange with meta-silicate, boric acid and borax was found to be complete in a short time at 100 °C, but the interchange was found to be somewhat slower with chromate and dichromate in neutral or basic solutions.

Jackman and Keenan found that the reaction between alkali metals and ammonia was catalyzed by borosilicate glass (Pyrex® and Kimax®).^135^ The glass vessels used were carefully cleaned by aqua regia, double distilled water followed by baking *in vacuo* at temperatures above 400 °C. The Brønsted and Lewis acidity of the borosilicate glass surface was suggested to be the active catalyst and especially the silanols were proposed to be crucial for the catalytic activity. When the silanols were removed by treating the surface with ammonium fluoride before baking, or potassium vapors, the activity decreased greatly. From their kinetic study, a mechanism was proposed, where the ammonia is bound to the acidic surface of the vessel. This, in turn, makes ammonia more acidic and consequently increases the rate of reaction with a solvated electron.

When the glass surface is found to promote a chemical reaction, it is not surprising that increased reaction rates can be achieved by increasing the glass surface area. A simple way of doing so is by adding powdered glass to the reaction. Gupta and coworkers found that glass powder, from transparent light bulbs, could be used as a catalyst for the auto-oxidation of sulfur dioxide. The glass was washed with acetone, left overnight in a dichromate solution followed by rinsing with water. In order to remove trace metal ions, the glass was washed with a combination of NaOH and ethylenediaminetetraacetic acid (EDTA) before a last washing with perchloric acid and water. Catalytic activity from metal ions leaching from the glass could be ruled out by trying to promote the reaction by glass-extracts, which showed the same reaction profile as the uncatalyzed reactions.

Sehested *et al.* found that the decomposition reaction of ozone in acidic solutions was dependent on the *in situ* formation of hydrogen peroxide to initiate the decomposition.^136^ The hydrogen peroxide could be formed on the surface and it was therefore studied whether there was a dependence between the surface area of the reaction vessels and reaction rate as the size of the borosilicate glassware employed varied in volumes from 25 mL to 5 L. Interestingly, a dependence of vessel volume and ozone decomposition could only be observed when hydrogen peroxide was added to initiate the process, even though hydrogen peroxide formation in the reaction was verified to take place at the surface. This observation could be explained by proposing that the termination reaction was also catalyzed by the vessel surface and hence larger vessels gave faster reactions as the volume-to-surface-area ratio became smaller.

The hydration of NO_2_ is believed to be a key source of nitrous acid (HONO) in the atmosphere. Barney and Finlayson-Pitts found that wet, porous borosilicate glass resulted in the formation of NO, N_2_O and HONO are formed in parallel with HNO_3_, which sticks to the surface.^137^ Their data suggests that glass surface catalyzes the hydration of NO_2_ and that N_2_O_4_ is an intermediate in this process. When studying the same reaction in Teflon® coated chambers, Pitts *et al.* found that 50% of the nitrogen could not be accounted for and that no HNO_3_ could be detected in the gas phase, which could be due to interaction with the vessel surface.^138^ Sakamaki *et al.* also found that the reaction between NO_2_ and water was presumably taking place on the surface of the smog chamber vessel.^139^ The exact role of the surface is not clear, but it seems like glass is not a prerequisite for the reaction to take place.

The influence of the vessel material on the self-assembly of metal suprastructures has been studied by Li *et al.*^140^ In regular washed glassware, only Ag nanowires were synthesized, whereas borosilicate glassware heavily cleaned with aqua regia gave rise to suprastructures from Ag nanoparticles. It was observed that washing the glassware once with aqua regia was not enough to achieve the suprastructures and it was therefore hypothesized that the vessel surface had changed properties upon the heavily washing. Surface roughening or functionalization was believed to influence the nucleation and growth of Ag nanoparticles, and this was supported by “decorating” the glass surface with different molecules, resulting in various morphologies depending on the vessel surface modifications.

## Conclusions

This review collects important results from the scientific literature or the past century which shows that the surface of regular laboratory glassware should not be considered inert during chemical reactions. It is evident that the vast majority of reported vessel effects concern organofluorine chemistry, but a significant number of reports involving Brønsted acid/base catalysis (most notably in recent reports by Cooks and co-workers^117,118^) should inspire our peers to probe the impact of vessel material as a standard variable during reaction development. In fact, we believe that the ramifications of vessel effects throughout the scientific literature are more far-reaching than has previously been reported, since vessel effects are typically not probed during reaction development or optimization. Given the numerous acid- and base-catalyzed chemical transformations, the fact that the surface of regular laboratory glassware is potentially capable of buffering the reaction medium might have a significant impact on such catalytic transformations as some of the catalyst is required to overcome the buffering effect of the vessel.

Given the literature precedence, we believe that there are reported chemical reactions that could significantly benefit from being carried out in an alternative vessel material. It is plausible that some reactions, especially in the field of organofluorine chemistry, have been abandoned or simply found to perform very poorly due to being conducted in regular laboratory glassware instead of in HF/fluoride-resistant reaction vessels.

The field of metal–organic chemistry has seen several examples of transition metal-catalyzed transformations at ppm levels of catalyst. Given how porous a glass surface can become over time because of solvolysis or glass cleaning procedures in strong base or acid, we believe that one should always be alert to the risk of having transition metal- or alkali metal impurities leaching from an old vessel into solution. Over the years, remarkable transition metal-catalyzed transformations have been reported to require only ppm to ppb (“homeopathic”) amounts of the active metal.^141–143^ In fact, such low quantities of metal catalysts can originate as an impurity from used stirbars,^144^ causing unexpected “phantom” transformations by unrecognized impurities in the reaction environment or reagents. By analogy with this, we postulate that there must be chemical reactions in the literature that are catalyzed or co-catalyzed either directly by the surface of a glass vessel or by catalytically active species released from the glass vessel during the reaction. We are unable to provide any suggestions as to how to best maintain regular laboratory glassware as either treating it with strong acid or base (as is common practice in many chemical laboratories) or even drying/silylation procedures will have specific consequences for the chemical and mechanochemical properties of the glass surface. The previous history of chemical reactions and cleaning procedures that a glass vessel has been exposed to can therefore impact the extent and nature of a given vessel effect. We do not know the exact consequences of this but wish to alert our peers to this phenomenon by the illustrated examples in this review.

## General considerations for vessel effects

Based on our review, we feel compelled to provide a brief list of suggestions on when and how to investigate vessel effects during chemical reactions.

As changing the vessel material can in principle have a positive or negative effect for a given reaction, we do not believe that fluorinated or non-fluorinated plastic polymers generally provide a superior vessel material to borosilicate glassware or other glass types. Hence, we would suggest implementing the following controls during reaction development:

### C–F bond activation under non-basic conditions

HF is frequently reported to be formed during C–F bond activation reactions that take place in the absence of base or an HF-scavenger. It is suggested to run parallel-experiments in an HF-resistant polymer material during reaction development to assess vessel effects.

### C–F bond formation

Some reagents used for C–F bond formation (especially XeF_2_) are very clearly influenced by the vessel material and parallel-experiments in HF-resistant polymers should be conducted during reaction development or optimization. Other reagents typically used for deoxyfluorination (*e.g.*, DAST, XtalFluor-E, *etc.*) should in theory be capable of generating HF during reactions and we would advise performing parallel experiments in HF-resistant vessels during such reactions.

### Storage of organofluorine compounds

It is well-described that certain organofluorine compounds have significantly longer shelf-lives in polymer-based containers compared to glass-based containers. Therefore, it is advisable to always store such compounds in polymer-based containers. Especially if C–F bond cleavage leads to formation of stabilized cationic intermediates, as glass seems to further accelerate the autocatalytic decomposition of organofluorine compounds. Especially benzylic and allylic fluorides seem to require polymer-based containers for storage. In our personal experience, glycosyl fluorides (that are otherwise remarkably stable) can suddenly decompose in glass containers after storage for extended periods of time, especially after being subjected filtration through filtration agents like Celite®, which potentially contaminates the product with small amounts of metal salts that can initiate the decomposition.

### Reactions catalyzed by strong Brønsted acid or base

As glass surfaces can have microfractures and increased porosity because of previous reactions or cleaning/pre-treatment conditions, various equivalents of silanols and boronic esters/acids could be exposed on the glass surface. These can influence a chemical reaction and it has even been reported that the glass surface itself can serve as a Brønsted base-catalyst. We recommend controlling for vessel effects during reactions using low catalyst loadings of either acid- or base catalysts (potentially both Lewis- and Brønsted acid/bases) by running parallel experiments in a plastic polymer reaction vessel.

## Author contributions

MMN and CMP wrote the manuscript and have approved the manuscript in its final form.

## Conflicts of interest

The authors declare no conflict of interest.

## Supplementary Material
